# The Intranidal Myrmecophiles of the Maltese Islands with Notes on *Messor* Nests as Repositories of Biodiversity

**DOI:** 10.3390/insects14010045

**Published:** 2023-01-03

**Authors:** Thomas Cassar, Albena Lapeva-Gjonova, David Mifsud

**Affiliations:** 1 Department of Biology, Faculty of Science, University of Malta, MSD 2080 Msida, Malta; 2 Department of Zoology and Anthropology, Faculty of Biology, Sofia University, 1164 Sofia, Bulgaria; 3 Institute of Earth Systems, Division of Rural Sciences and Food Systems, University of Malta, MSD 2080 Msida, Malta

**Keywords:** myrmecophily, myrmecology, harvester ants, symbiosis, ant nests, Mediterranean, Malta, ar-thropods, new records, associations

## Abstract

**Simple Summary:**

Ant nests contain a multitude of resources which some organisms have evolved to exploit, living in close association with ants in order to prey on them, feed on their stored food, or simply in order to make use of their closely guarded fortresses as safe shelters. These organisms, known as myrmecophiles, have never been studied in much detail in the Maltese Islands—a small Mediterranean archipelago which, despite being one of the most densely populated countries in the world and facing enormous human pressures, has been shown to harbour a great diversity of insects, arachnids and crustaceans. In this study, we aimed to catalogue Maltese myrmecophile diversity and provide notes on the biology of all species encountered. As a result, we found at least thirty different species of insect, arachnid and crustacean which live exclusively inside ant nests in the Maltese Islands, four of which had never been known to inhabit this archipelago. An aphid-ant and a spider-ant association also appear to be previously unknown. We also discuss how the nests of *Messor* harvester ants may be important biodiversity hotspots due to these ants’ nests being suitable homes for many different species when compared to the nests of other types of ant.

**Abstract:**

The intranidal myrmecophilous arthropod fauna of the Maltese Islands is reviewed. Thirty species from nine orders are found to be obligate myrmecophiles, of which four species are recorded from the Maltese archipelago for the first time: *Phrurolithus* sp. (Araneae: Phrurolithidae), *Pogonolaelaps canestrinii* (Berlese, 1904), *Gymnolaelaps messor* Joharchi, Halliday, Saboori & Kamali, 2011 and *G. myrmecophilus* (Berlese, 1892) (Mesostigmata: Laelapidae). *Phrurolithus* also represents the first record of the family Phrurolithidae in Malta. Notes on the biology and local distribution of each species are provided, including ant-myrmecophile associations, of which two appear to be previously unknown: the occurrence of *Smynthurodes betae* Westwood, 1849 (Hemiptera: Aphididae) in the nest of *Plagiolepis pygmaea* (Latreille, 1798) and *Phrurolithus* in the nest of *Pheidole pallidula* (Nylander, 1849). Fourteen additional species are found to be either only occasionally myrmecophilic, accidental ant-guests or potentially myrmecophilous, the latter remaining ambiguous due to a lack of knowledge of their biology. Of these, the family Caeculidae (Arachnida: Trombidiformes) represents a new record for the Maltese Islands, on the basis of *Microcaeculus* sp. occurring in a nest of *Camponotus barbaricus* Emery, 1905. Preliminary results indicate that *Messor* nests may be repositories of considerable myrmecophile diversity, with the most unique symbionts.

## 1. Introduction

Impregnable as they may seem, ant nests are incredibly rich microhabitats: bounteous nutritive resources in the form of defenseless larvae, harvested food and piles of refuse; a finely regulated microclimate in which humidity and temperature rarely stray outside a range of tolerance; and above all else a shelter from marauding predators, guarded by a battery of mandibles, envenomating stings or explosively propelled formic acid [1]. An incredibly diverse array of arthropods have, through physio-morphological and behavioural adaptation, evolved to exploit ant nests for these resources: the myrmecophiles [2]. Throughout the history of myrmecophilous studies, the term has come to characterize a broad range of interactions between ants and other organisms [3]. By far the most specialized organisms in this regard are those which have taken up a life completely within the nests of ants: the intranidal myrmecophiles. Here, they may live as commensal inquilines which the ants pay little attention to; others live in reciprocating relationships with their hostesses, earning their keep by providing products or services; whilst others are outright predators, parasites or parasitoids of the ants, making use of various mechanisms to remain undetected and unharmed.

The upper estimate for global myrmecophile diversity stands at 100,000 species, though only 10% of these species are obligate myrmecophiles [4]. Today, a wide range of arthropod taxa are known to include myrmecophilous species, such as beetles [5], true bugs and their allies [6,7,8,9], cockroaches [10,11], crickets [12], true flies [13,14], parasitoid wasps [15], moths and butterflies [16,17], silverfish [18,19], springtails [20,21], spiders [22], pseudoscorpions [23,24], mites [25,26,27,28] and even woodlice [29]. Each myrmecophilous arthropod has become adapted for a life in the nest chambers of ants, and such adaptations are missing from arthropods whose presence in ant nests is more or less accidental due to their soil-inhabiting nature. Myrmecophilous arthropods may be morphologically adapted for phoresy, ectoparasitism, myrmecomorphy or sound mimicry [25,30,31,32,33,34,35]. Other species may employ chemical mimicry, either synthesizing ‘nest odour’ chemical cues de novo or acquiring them gradually through direct contact with their hosts [36,37,38].

The Maltese Islands are situated in the centre of the Mediterranean Sea, aligned in a North-West to South-East direction. The total area of the archipelago amounts only to 316 km^2^, and they lie some 96 km to the south of Sicily and some 350 km north of the Libyan coast. Malta, Gozo and Comino—the three largest islands of the archipelago—are inhabited, with a collective population of almost 520,000 people and the fifth-highest population density of any country in the world at 1649 persons per square kilometre. A number of uninhabited islets and rocks also occur along the coast. The climate is typically Mediterranean, with hot, dry summers and mild, wet winters. Despite enormous human pressures on the natural environment of the Islands, they are home to an estimated 4500 species of terrestrial and freshwater arthropods, and hundreds of species continue to be added to the archipelago’s known fauna—including various newly described taxa [39,40,41,42,43]. Such burgeoning island biodiversity paired with rapid urban encroachment into natural habitats has great implications for conservation.

Despite a growing interest in the study of Central Mediterranean invertebrate diversity, the myrmecophilous arthropods inhabiting the Maltese Islands have not yet received much attention. Although various species which are known to be myrmecophilous have been recorded from the archipelago, such records usually occur as part of broader taxon-wide studies and faunal reviews, and do not include much ecological data such as ant-host associations. *Dichillus pertusus* (Kiesenwetter, 1861)—a myrmecophilous tenebrionid—is fleetingly mentioned in a list of beetles from Malta, but no information is provided to accompany the record besides a vague location [44]. *Cyphoderus albinus* (Nicolet, 1842), a myrmecophilous springtail, is included in a list of Maltese collembolans—but only as being found “under flower-pots” [45]. *Italochrysa italica* (Rossi, 1790) is briefly mentioned from Buskett as part of a broader work on Maltese chrysopids; its larvae are known to inhabit the nests of *Crematogaster* [46]. A tettigometrid was found “attended by *Camponotus*” in Malta, but the material presented was not taken from within ant nests—some tettigometrids are known to live inside the colonies of *Lasius*, *Tapinoma* and *Tetramorium* [47].

The only exception to this paucity of information is the case of the ant-loving crickets (*Myrmecophilus* spp.) and that of the myrmecophilous platyarthrid isopods. Since its first mention from the Islands and the description of a new species [48], the genus *Myrmecophilus* has received much attention by various authors treating the distribution and host associations of these crickets in the Maltese Islands [49,50,51,52,53]. The myrmecophilous isopod family Platyarthridae has also been given relatively more attention than other ant-associated arthropod taxa, and ant-nest associations and distribution for all species in the Maltese Islands have been relatively well-studied [54,55,56]. It should also be noted that a broader work of the Maltese myrmecofauna mentions three myrmecophilous hemipterans which were encountered in ant nests, including host associations for the aphid *Trama baronii* (Hille Ris Lambers, 1969), the planthopper *Tettigometra impressifrons* Mulsant & Rey, 1855 and the scale insect *Lacombia dactyloni* (Bodenheimer, 1943) [57].

Presumably, with an estimated terrestrial arthropod diversity of 4500 species and more than seventy species of ant, the Maltese Islands harbor a great diversity of myrmecophilous taxa [39,58]. The paucity of local literature on many aspects of myrmecophily, however, stands in the way of gaining a true understanding of this diversity. The present works aims to provide the first account of the diversity of intranidal (within nest) myrmecophilous arthropods in the Maltese Islands, with notes on their distribution and ecology, through sampling of ant nests from a variety of locations across the archipelago and thorough examination of the literature.

## 2. Materials and Methods

**Figure 1:**
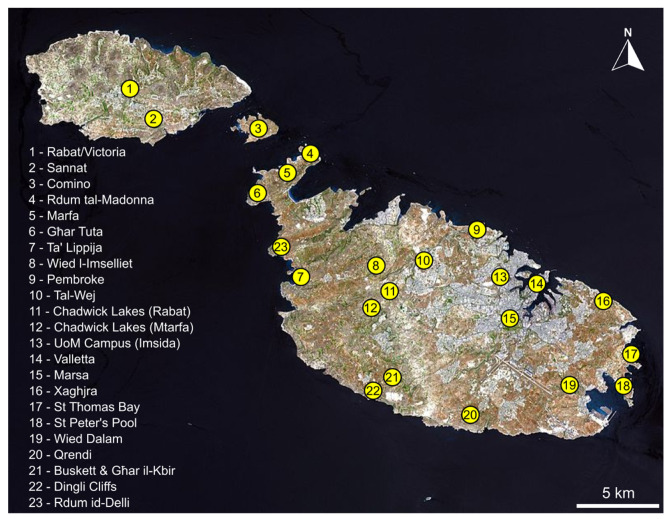
Map showing location of sampling sites for intranidal myrmecophiles across the Maltese Islands.

**Figure 2:**
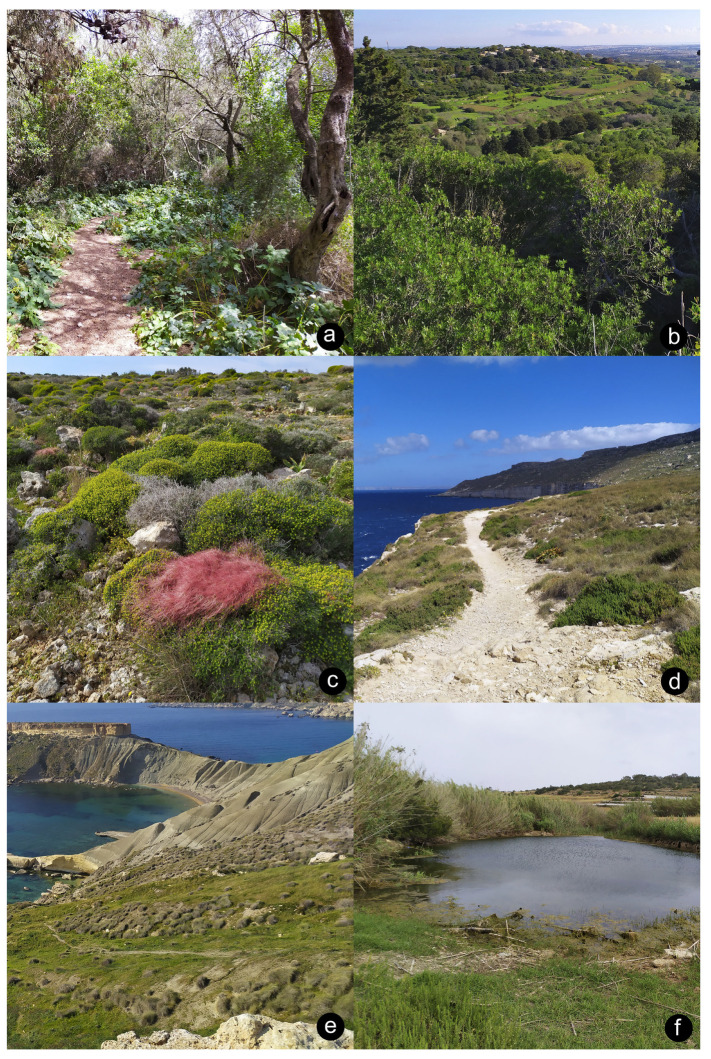

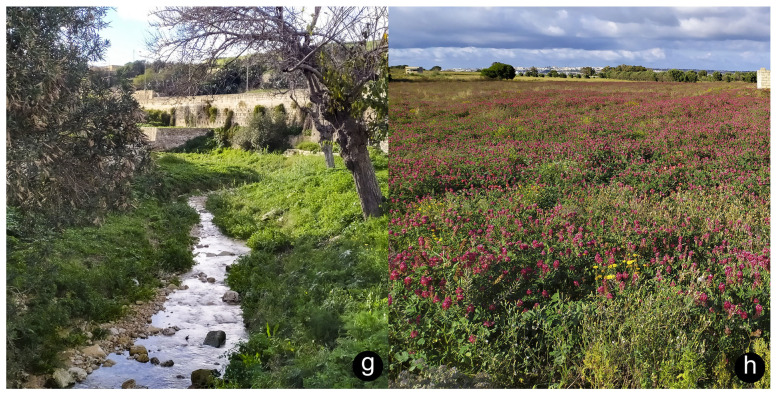
**Sampled habitats in the Maltese Islands.** (**a**) Semi-natural woodland, Buskett; (**b**) mosaic of maquis and agricultural land, Buskett; (**c**) garigue/phrygana, Marfa, Mellieħa; (**d**) coastal cliff plateau with sandy soil and low-growing vegetation, Miġra Ferħa, Rabat; (**e**) coastal clay slopes with steppe, Qarraba and Ġnejna Bay, Mġarr; (**f**) valley freshwater pool with loamy banks vegetated by *Arundo* and *Paspalum*, Imselliet Valley; (**g**) valley freshwater stream with gravel and diverse herbaceous vegetation, Lunzjata Valley, Gozo; (**h**) agricultural field growing animal fodder, Żebbuġ.

Ant nests were located by overturning rocks, fallen branches and other debris in a variety of habitats in twenty-three locations across Malta, Comino and Gozo (Figure 1 and Figure 2). First, a visual inspection of the exposed nest was carried out, collecting any visible myrmecophilous arthropods by using an aspirator (pooter). Then, the soil clinging to the rock/branch which was lying on top of the nest was scraped into a plastic white tray (30 cm × 24 cm × 8 cm) which was shaken periodically to expose any animals, collecting any myrmecophilous arthropods with an aspirator. Similarly, soil from within the nest chambers themselves was extracted with a trowel and subjected to the same tray-aspirator method. If the soil was too compacted to be sampled by the trowel, the soil constituting the nest was first broken up and loosened by striking it a few times with a small garden hoe, followed by extraction with a trowel. The nest of exceptionally small ants were sampled in this way but also subjected to Berlese-Tullgren funnel extraction in order to avoid missing any small myrmecophiles. A few specimens of the host ants themselves were also taken from each sampled nest in order to provide information about specific associations. All material was conserved in 80% ethanol. Each nest sample was designated a unique alphanumeric code corresponding to its particular collection data in order to keep track of which species were found together in the same nests and to provide information about host-ant associations for different myrmecophiles. Specimens are retained within the private collections of those who identified material (see Acknowledgments) as well as the authors’ private collections.

An extensive literature search was also conducted in order to determine which potentially myrmecophilous arthropods have been previously recorded from the Maltese Islands, even if their myrmecophilous nature was not explicitly stated. The literature search made use of Google Scholar and the University of Malta’s HyDi and Open Access Repository; volumes of the Bulletin of the Entomological Society of Malta, Potamon, Animalia, The Central Mediterranean Naturalist, The Maltese Naturalist, Bollettino della Società Entomologica Italiana and a number of other journals with potential of finding Maltese records of myrmecophiles were obtained and searched for any reference to taxa from Malta which are known to be myrmecophilous.

An annotated species list of intranidal myrmecophiles is provided in the results. “Previous records” refer to sources in which a particular species has already been recorded from the Maltese Islands. Arthropods encountered inside ant nests but which do not represent obligate myrmecophiles are listed in the Appendix A (Table A2).

## 3. Results

### 3.1. Insecta Linnaeus, 1758

#### 3.1.1. Orthoptera Latreille, 1793: Myrmecophilidae Saussure, 1874

***Myrmecophilus ochraceus* Fischer, 1853**

**Material examined.** MALTA: 1♀ (adult), Belvedere Il-Kalanka, 35°49′28.329″ N 14°33′23.399″ E, 02.06.2019; with *Messor capitatus*, leg. A. Lapeva-Gjonova; 3♂♂ (1 adult, 2 nymphs), Qrendi, 35°49′27.98″ N 14°26′54.76″ E, 29.II.2020, with *M. capitatus*, leg. T. Cassar; 3♂♂ (2 adults, 1 nymph) & 2♀♀ (1 adult, 1 nymph), same location, 16.V.2021, with *M. capitatus*, leg. T. Cassar; 1 nymph (sex indet.), same data, with *Tetramorium* sp. (*semilaeve* group), leg. T. Cassar; 2 nymphs (sex indet.), Qrendi, 35°49′28.50″ N 14°26′45.94″ E, 16.V.2021, with *M. capitatus*, leg. T. Cassar; 3♂♂ (1 adult, 2 nymphs), Pembroke, 35°55′58.42″ N 14°28′42.96″ E, 16.II.2020, with *M. capitatus*, leg. T. Cassar; 1♂, Msida, University of Malta Campus, 35°54′6.15″ N 14°29′4.47″ E, 5.V.2021, with *M. capitatus*, leg. E. Cutajar; 1 nymph (sex indet.), Rabat, Chadwick Lakes, 35°53′42.04″ N 14°23′37.22″ E, 25.VII.2021, with *Pheidole pallidula*, leg. T. Cassar; 1♂, Misraћ Gћar Il-Kbir (l/o Siġġiewi), 35°51′12.11″ N 14°23′52.85″ E, 29.IX.2019, with *M. capitatus*, leg. T. Cassar. COMINO: 3♂♂ (1 adult, 2 nymphs) & 4♀♀ (2 adults, 2 nymphs), 36°00′45.6″ N 14°20′07.0″ E, 23.II.2020, with *M. capitatus*, leg. T. Cassar. GOZO: 10 ♀♀ (8 adults, 2 nymph), Xewkija, Ġnien Ta’ Blankas, 36°1′43.038″ N 14°15′26.105″ E, 6.VI.2019, with *M. capitatus*, leg. A. Lapeva-Gjonova; 1♀, Sannat, Ta’ Ċenċ, 36° 1′15.63″ N 14°15′31.81″ E, 4.I.2021, with *M. capitatus*, leg. T. Cassar; 2♂♂ & 2♀♀, Rabat, 36°2′25.34″ N 14°14′39.48″ E, 28.II.2020, with *M. capitatus*, leg. B. Grech; 1♂, same location and collector, 21.II.2020, host unknown.

**Previous records**. [48,49,51,53,59,60,61].

**Global distribution.** Circum-Mediterranean (Morocco, Spain to Greece, Lebanon) [53].

**Local distribution and frequency.** Widespread and common; the following records are an amalgamation of a previous work [51] and the present study, as more recent works are reviews which do not present new material. In Malta it has been recorded from Gћajn Riћana, Gћar Lapsi, Qrendi, Gwardamangia, Lija, Manoel Island, Marsaxlokk, Mellieћa, Mistra, Paradise Bay, Spinola, St. Paul’s Bay, St. Thomas Bay, Wied Babu, Wied Qannotta, Wied is-Sewda, Pembroke, Msida and Chadwick Lakes. In Gozo it has been recorded from Ħondoq ir-Rummien, Mġarr, Ramla l-Ħamra, Qbajjar, San Lawrenz, Rabat, Xlendi and Sannat. On Comino it has been recorded near Saint Mary’s Tower, inland from San Niklaw bay, and in the central area near the old pumping station.

**Ecology.** As with other myrmecophilids, *M. ochraceus* can be considered a synoekete in Wasmannian terms, inhabiting the periphery of the nest structure and treated with indifference by its host ants. In the present study, these crickets were always collected through visual inspection of the stone covering the ant nest, never from soil extraction, indicating that the insects do not infiltrate the deeper nest chambers. Adults were always collected inside the nests of *Messor capitatus* (Latreille, 1798), but nymphs were sometimes found with smaller species, namely *Tetramorium* cf. *semilaeve* and *Pheidole pallidula* (Nylander, 1849). This species has been recorded as living with *Monomorium subopacum* (Smith, 1858), as well as *Tetramorium caespitum* (Linnaeus, 1758) and *Messor structor* (Latreille, 1798) locally [51], though the latter two likely do not occur in Malta and probably refer to other species. Multiple crickets were often found in the same nest in close proximity, especially if the nest was particularly large, confirming past observations that *M. ochraceus* is a gregarious myrmecophile.

**Remarks.** Some collected nymphs were too young to be completely certain of their identity. They are nevertheless included with the above material as they are likely to belong to this species as they were collected from nests in close proximity to other nests which hosted identifiable specimens *M. ochraceus*.

***Myrmecophilus baronii* Baccetti, 1966**

**Material examined.** None.

**Previous records.** [48,51,53,59,61,62,63].

**Global distribution.** Central-South Mediterranean (Malta, Pantelleria and Tunisia) [53].

**Local distribution and frequency.** Rare. Recorded from Buskett and St. Thomas Bay [51].

**Ecology.** As with other myrmecophilids, *M. baronii* can be considered a synoekete in Wasmannian terms, inhabiting the periphery of the nest structure and treated with indifference by its host ants. This species has been recorded as usually living with *Camponotus barbaricus* Emery, 1905, though occasionally being found with *Messor* (?) *structor* [51]. In Tunisia, this species has also been collected with an unidentified *Camponotus* species of the subgenus *Tanaemyrmex*; here they were collected in pine forests or stony, open areas [64]. Type material for its original description as well as more recent material from elsewhere in the Mediterranean consists of multiple individuals from the same location, which may indicate that *M. baronii* is also a gregarious myrmecophile [48,64].

***Myrmecophilus fuscus* Stalling, 2013**

**Material examined**. None.

**Previous records**. [52,53].

**Global distribution**. Euro-Mediterranean (Spain eastward to Croatia and southward to Malta) [52].

**Local distribution and frequency**. Only recorded from Buskett [52,53].

**Ecology**. As with other myrmecophilids, *M. baronii* can be considered a synoekete in Wasmannian terms, inhabiting the periphery of the nest structure and treated with indifference by its host ants. This species has been recorded from the nests of *Crematogaster scutellaris* (Olivier, 1792) in dead tree trunks and branches, as well as in the nests of *Lasius, Camponotus* and *Formica* species [65]. No recorded ant hosts are mentioned for the single specimen collected in Malta [53].

***Myrmecophilus quadrispinus* Perkins, 1899**

**Material examined**. MALTA: 1♂, Żebbuġ, 2019, in private residence, photographed by T. Cassar; 5♂♂ & 1♀, Paola, 2020–2021, in private residence, leg. D. Mifsud.

**Previous records.** [66].

**Global distribution**. Hawaii, New Caledonia, Japan (Nansei Is., Ogasawara Is.), Mauritius, Samoa, Taiwan, Malta, Peru [66,67,68].

**Local distribution and frequency**. So far, the only confirmed records of this species come from Żebbuġ and Paola (see Remarks).

**Ecology**. *M. quadrispinus* is considered a cryptogenic tramp species—it has been found in city gardens, under stones alongside anthropogenically disturbed areas and among ornamental plants being transported by boats [67,69]. This, together with its tendency to occur with invasive ants such as *Anoplolepis gracilipes* (Smith, 1857)*, Paratrechina longicornis* (Latreille, 1802)*, Solenopsis* and *Pheidole*, suggests that *M. quadrispinus* is introduced into new territories alongside and through the same pathways as its ant hosts, primarily as a result of human commerce [67]. Its exclusive occurrence inside human homes in Malta as observed in the present study conforms to this notion, though its local ant hosts so far remain unknown. It has also been recorded in association with *Carebara, Polyrhachis, Nylanderia, Camponotus, Diacamma* and *Brachyponera*, and is a generalist species which inhabits the nests of both native and non-native ants wherever it is introduced [67]. This species has poor host-mimicry capabilities, appears to be unable to obtain the cuticular hydrocarbons of ants and must stave off frequent aggressive interactions from its hosts simply by swiftly running away [69,70].

**Remarks**. This species may also occur in Żurrieq (Wied Babu area) and Balzan, as photographs which may correspond to this species have been uploaded to social media by homeowners seeking identification. The photographs are not of sufficient quality to be certain of the crickets’ identity. It is nevertheless noteworthy that in all cases—confirmed and unconfirmed sightings—the crickets were encountered scurrying about on the surface of tiled floors inside private residences.

#### 3.1.2. Hemiptera Linnaeus, 1758: Tettigometridae Germar, 1821

***Tettigometra atra* Hagenbach, 1822**

**Material examined**. MALTA: 1 ex., Baћrija, 35°53′44.4″ N 14°21′04.7″ E, 2015, under rock, leg. T. Cassar.

**Previous records**. [47,71].

**Global distribution**. Widely distributed throughout the Palaearctic; recorded from much of Europe; North Africa; Western to Central Asia (Armenia east to Mongolia) [47].

**Local distribution and frequency**. Not uncommon and possibly quite widespread as it has been recorded from Birżebbuġa, St Thomas Bay and Baћrija [47].

**Ecology**. Adults usually feed on lower parts and roots of both herbaceous and woody vegetation; specimens have been recorded feeding on the roots of *Reichardia picroides* (L.) Roth and being attended to by *Camponotus barbaricus* due to their secretion of honeydew [47]. No specimens have been taken from inside ant nests themselves in Malta, but *Tettigometra atra* is known to occur inside the nest structure of *Lasius* and *Tetramorium* elsewhere [72].

**Remarks**. The above mentioned material was collected under a rock, which may have been in fact atop an ant’s nest, but at the time the collector was not sampling for myrmecophiles, and thus it cannot be ascertained in retrospect which species of ant, if there were any, the hopper was living with.

***Tettigometra impressifrons* Mulsant & Rey, 1855**

**Material examined**. MALTA: 1♀, Paradise Bay, 35°58′51.425″ N 14°20′0.693″ E, 19.XI.2017, with *Tapinoma simrothi*, leg. A. Lapeva-Gjonova. COMINO: 1 ex., central area, 36°00′45.6″ N 14°20′07.0″ E, 23.II.2020, with *T. simrothi*, leg. T. Cassar.

**Previous records**. [57,71].

**Global distribution**. Widely distributed around the Mediterranean—North Africa, Western Asia, Southern and Western Europe [71].

**Local distribution and frequency**. Uncommon but, though under-recorded, appears to be widespread as it has been recorded from Żejtun [71] and now—for the first time—from the island of Comino.

**Ecology**. This species has been observed living inside the nests of *Tapinoma* on the sandy coasts of Apulia and Sicily, sometimes occurring with its congener *Tettigometra griseola* Fieber, 1865 (Gjonov, *pers. comm*., 2021). Its occurrence in a nest of *Tapinoma simrothi* Krausse, 1911 on Comino seems to confirm its apparent preference for *Tapinoma* nests in the Central Mediterranean. This species has been found to be very frequent in nests of *Tapinoma erraticum* (Latreille, 1798) [57]. Presumably, as in other *Tettigometra* species in Europe, the leafhoppers are kept inside the nests by the ants themselves during the winter months in order to protect them, as they are sources of nutritive honeydew for their hosts [73].

**Remarks**. This species is possibly tended to by ants outside the nest on foliage, but then carried back inside the nest structure by its hosts for ‘safekeeping’ during the winter months. Thus it is a myrmecophile associated with the nest itself, albeit only seasonally.

***Tettigometra laeta* Herrich-Schäffer, 1835**

**Material examined**. None.

**Previous records**. [71].

**Global distribution**. Mostly Mediterranean, occurring throughout much of southern Europe and North Africa, though it occurs in the southern parts of some Central European countries as well [71].

**Local distribution and frequency**. Appears to be a rather uncommon species; it has been recorded from the Verdala Palace grounds in Buskett and Ta’ Qali National Park in Attard [71].

**Ecology**. *Tettigometra laeta* has been observed in the nests of *Lasius psammophilus* Seifert, 1992, *Tetramorium caespitum* and *Formica cunicularia* Latreille, 1798 in the coastal sand dunes of Belgium [73]. Apart from being taken into the nest by their ant hosts upon disturbance, it has been suggested that the planthoppers may also develop as nymphs within the protection of the ant nest structure [73].

#### 3.1.3. Hemiptera: Aphididae Latreille, 1802

***Trama baronii* (Hille Ris Lambers, 1969)**

**Material examined**. None.

**Previous records**. [74,75].

**Global distribution**. Malta, Italy.

**Local distribution and frequency**. Collected from Mellieћa Bay once in the 1960s and seemingly never recorded again, indicating that this species may either be rare or under-collected [57].

**Ecology**. Referring to this species as ‘*Protrama urbanii*’ prior to the publication of its formal description, Baroni Urbani writes the following in Italian: “Aphids collected in the nests of *Camponotus barbaricus* were … feeding on the roots of *Carduus* sp. … The ants had, towards [*Trama baronii*], a behaviour similar to that reserved for other true myrmecophiles, and they quickly transported the aphids into the deepest tunnels of the nest upon disturbance” [57]. Thus it can be assumed that this species is a root-feeding trophobiont which exhibits mutualism with *Camponotus*.

***Rectinasus buxtoni* Theobald, 1914**

**Material examined**. COMINO: 5 apterae, 36°0′50.53″ N 14°19′48.24″ E, 23.II.2020, underside of rock on top of *Pheidole pallidula* nest, leg. T. Cassar.

**Previous records**. [76].

**Global distribution**. Southern Europe and North Africa, South-West and Central Asia [76].

**Local distribution and frequency**. Appears to be a rare and localized species, so far only recorded from southern Gozo [76] and now from the island of Comino. However, this may simply represent a lack of thorough collection efforts.

**Ecology**. The primary hosts for this species are various *Pistacia*, especially *P. khinjuk* Stocks, 1852 and *P. terebinthus* L., on which it creates long, spindle-shaped leaf galls and undergoes a hetereoecious holocycle with sexually reproducing males and females [77]. However, in the western Palaearctic where most of its favoured *Pistacia* primary hosts are not present, the aphid tends to occur on the roots of various secondary hosts such as a number of Poaceae and Asteraceae, where it is anholocyclic, producing only females through parthenogenesis [78]. Its occurrence in the nests of ants is due to its preference for the roots of its secondary hosts, though the relationship between the aphids and their formicid hosts appears not be fully understood [79]. A trophobiotic relationship is assumed, as the aphids are tolerated in the ants’ nest; in the present study the aphids were encountered as an aggregation on the underside of a rock with *Pheidole pallidula*, where they were not treated with any hostility by their hosts.

***Smynthurodes betae* Westwood, 1849**

**Material examined**. MALTA: 1 aptera, Mellieћa, Marfa, 35°58′54.39″ N 14°21′6.04″ E, 15.II.2020, in soil with *Plagiolepis pygmaea*, leg. T. Cassar.

**Previous records**. [76,80,81].

**Global distribution**. Extremely widely distributed; range includes most of Eurasia, North Africa and the Afrotropical region [81].

**Local distribution and frequency**. It has been collected from Marfa and San Anton Gardens in Malta, as well as Żebbug in Gozo [80,81]. This suggests that the species is relatively widespread locally.

**Ecology**. This species usually undergoes a host-alternating life cycle, initially causing leaf galls on a number of *Pistacia* species, especially *P. atlantica* Desf., 1799 and *P. terebinthus*, later emerging and migrating to the roots of various dicots [77]. When feeding on the roots of these plants, they are often attended to by ants, and may occur in ant nests built around plant roots such as with *Lasius*, *Solenopsis* and *Tetramorium* [82]. Though this aphid may be anholocyclic when its primary host is not available, the populations in the Maltese Islands may be assumed to undergo a hetereoecious holocycle as the species has been found both causing galls on *Pistacia atlantica* [81] and in soil around roots with ants (present study). The association between *S. betae* and *Plagiolepis pygmaea* Latreille, 1798 appears to be a previously unrecorded ant-aphid association.

#### 3.1.4. Hemiptera: Pseudococcidae Heymons, 1915

***Lacombia dactyloni* (Bodenheimer, 1943)**

**Material examined**. MALTA: Pembroke, 35°55′58.42″ N 14°28′42.96″ E, 2.IV.2021, with *Tapinoma simrothi*, leg. T. Cassar; Dingli Cliffs, 35°50′57.05″ N 14°23′24.51″ E, 22.II.2020, with *T. simrothi*, leg. T. Cassar. COMINO: 36°00′45.6″ N 14°20′07.0″ E, 23.II.2020, from four separate nests of *T. simrothi*, leg. T. Cassar; 36° 0′50.53″ N 14°19′48.24″ E, 10.IV.2021, from two separate nests of *T. simrothi*, leg. T. Cassar.

**Previous records**. [83,84].

**Global distribution**. Mediterranean: Malta, Israel, Tunisia [85].

**Local distribution and frequency**. Appears to be quite widespread, collected from Dingli, Pembroke, Birzebbuġia and the island of Comino [83,84], [present study].

**Figure 3:**
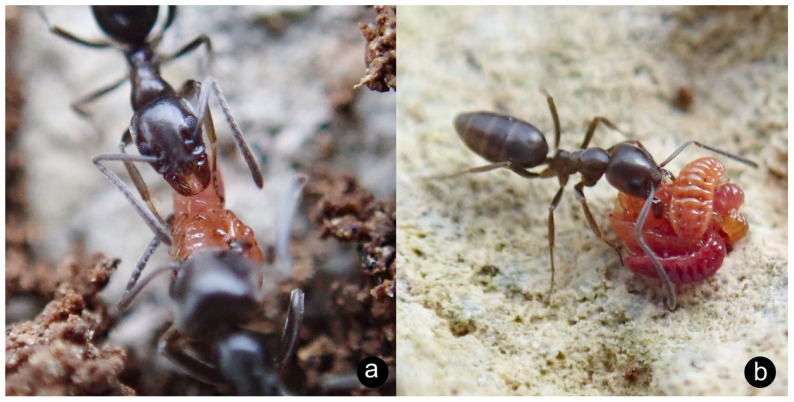
*Lacombia dactyloni* individuals carried by *Tapinoma* after lifting rock off nest, Dingli; (**a**) individuals being transferred from one worker to another; (**b**) individuals carried as a spherical mass by a worker.

**Ecology**. A trophobiont, this species feeds on the roots of various Poaceae and Asteracae inside the nests of *Tapinoma erraticum* [83]. Recorded host plants include *Artemisia, Anacyclus, Cynodon* and, in Malta, *Chiliadenus bocconei* Brullo [84,85]. In the present study, *L. dactyloni* was collected exclusively from the nests of *Tapinoma simrothi* by scraping the underside of rocks lying on top of the nest or collecting individuals from the soil of the upper chambers of the nest (Figure 3a,b).

**Remarks**. The taxon occurring in the Maltese Islands was initially described as a new species, *Lacombia urbanii*, named after Baroni Urbani who collected it from *Tapinoma* nests on Comino in great numbers [68,83]. However, it was later synonymized with *Lacombia dactyloni* [86].

#### 3.1.5. Neuroptera Linnaeus, 1758: Chrysopidae Schneider, 1851

***Italochrysa italica* (Rossi, 1790)**

**Material examined**. MALTA: 2 exs., Buskett, 22.VII.2019, leg. J. Farrugia & J. Formosa.

**Previous records**. [46,87,88].

**Global distribution**. Circum-Mediterranean [88].

**Local distribution and frequency**. Present at Buskett, where adults can be observed in low numbers during the months of July and August [46].

**Ecology**. Though the large and distinctively-coloured adults are not myrmecophilous, the larvae of *I. italica* are associated with *Crematogaster scutellaris*. Adults lay eggs borne on long, silk threads during July and August, attaching them within close proximity of *C. scutellaris* nests—usually trees and wooden structures [89]. Immediately after hatching, the larvae begin to attach pieces of tree bark and soil particles to the bristles covering their body, effectively creating a case of armour around themselves. They then wander through cracks and crevices around the ant nest, often entering deep into the nest itself, where they are usually ignored by their hosts due to their armour of debris [89]. Inside the nests of *Crematogaster*, the lacewing larvae position themselves in high-traffic tunnels, where they lie in ambush as workers stream past, lunging forward only to snatch larvae and pupae being carried in the jaws of workers. Ant larvae and pupae are then ‘sucked dry’ [89]. The larvae of *I. italica* do not feed on the workers themselves, instead capturing them and holding them close to their debris-armour and then releasing them; probably in order to obtain nest odour [90].

**Remarks**. The adult specimens examined in the present study were collected in Buskett, as were Anthony Valletta’s in 1984 [46], and though no larvae were encountered in *C. scutellaris* nests, this ant species is incredibly abundant in Buskett (*pers. obs.* T. Cassar) and it is not unlikely that much the same ecology for this species as observed elsewhere in the Mediterranean applies to the Maltese Islands as well.

#### 3.1.6. Coleoptera Linnaeus, 1758: Tenebrionidae Latreille, 1802

***Dichillus pertusus* (Kiesenwetter, 1861)**

**Material examined**. MALTA: 1 ex., “G.C. Champion coll. BM 1927-409”.

**Previous records**. [44,91,92].

**Global distribution**. Italy (including Sicily, Pantelleria and the Egadi Islands), Greece and Anatolia [93].

**Local distribution and frequency**. Probably locally extinct; a single confirmed record was made on the basis of specimens collected in Valletta well over a century ago [44].

**Ecology**. This species has been recorded in association with various species of ants which nest under stones [44]. What the beetle feeds on within the ant nest, or any other aspects of its ecology, appear to be unknown.

**Remarks.***Dichillus pertusus* was recorded from the Maltese Islands for the first and only time over a century ago as “very rare”, with solely three specimens collected from “Porto Reale” [44]. The specimens were later examined and confirmed to be correctly identified, leaving no doubt that the species was indeed once present in Malta [91]. However, in the 115 years since their first record, no other specimens of *D. pertusus* have been collected from Malta. The present authors conducted an extensive search of the area in Valletta to which “Porto Reale” would correspond to, during a phenologically favourable time for this species (May), but no specimens were found, even though ant nests were present. It is therefore apparent that this species is locally extinct.

#### 3.1.7. Coleoptera: Endomychidae Leach, 1815

***Cholovocera fleischeri* (Reitter, 1902)**

**Material examined**. MALTA: 3 exs., St Thomas Bay, 8.V.1997, leg. D. Mifsud; 4 exs., Buskett, 4.V.1997, leg. D. Mifsud; 1 ex., Tal-Munxar, 17.V.1997, leg. D. Mifsud; 1 ex., “Karaba”, 13.I.1999, leg. D. Mifsud; 1 ex., Balluta (Marsaxlokk), 26.X.1996, leg. D. Mifsud; 2 exs., Mellieћa, Gћar Tuta, 35°58′36.91″ N 14°19′42.13″ E, 13.X.2019, with *Messor capitatus*, leg. T. Cassar; 3 exs., Pembroke, 35°55′58.42″ N 14°28′42.96″ E, 16.II.2020, with *Tapinoma simrothi* & *M. capitatus*. GOZO: 1 ex., Dwejra, 16.X.1997, leg. D. Mifsud; 2 exs., Victoria, 28.X.1995, in ant nest, leg. D. Mifsud; 1 ex., Xewkija, Ġnien Ta’ Blankas, 36°1′43.038″ N 14°15′26.105″ E, 6.VI.2019, with *M. capitatus*, leg. A. Lapeva-Gjonova; 1 ex., Sannat, Ta’ Ċenċ, 36°1′15.63″ N 14°15′31.81″ E, 4.I.2021, with *M. capitatus*, leg. T. Cassar. COMINOTTO: 1 ex., 5.V.1990, leg. D. Mifsud.

**Previous records**. [94,95].

**Global distribution**. Italy, Malta, Montenegro, Serbia, Croatia [95,96].

**Local distribution and frequency**. Appears to be a common, widespread species recorded from southeastern to northwestern Malta, Cominotto and northwestern-central Gozo.

**Ecology**. Little is known about the specific ecology of *C. fleischeiri*. Presumably, like other Mediterranean species of the genus *Cholovocera*, it is an obligate nest-dweller of *Messor* species [97]. The beetle was found “under stones in an unidentified ant’s nest” and “in a willow stump with unidentified ants” in southern Italy [96].

***Cholovocera formicaria* (Motschulsky, 1838)**

**Material examined**. MALTA: 6 exs., Mellieћa, Paradise Bay, 28.XI.1993, leg. D. Mifsud; 8 exs., Mosta, Wied il-Gћasel, 25.XI.1993, leg. D. Mifsud; 2 exs., Marsaxlokk, Xrobb l-Għaġin, 27.XI.1993, leg. D. Mifsud; 2 exs., Manoel Island, 2.VI.1990, leg. D. Mifsud; 12 exs., Dingli, Buskett Gardens, 35°51′21.781″ N 14°23′52.244″ E, 3.VI.2019, with *Messor capitatus*, leg. A. Lapeva-Gjonova; 2 exs., Mellieћa, Rdum Tal-Madonna, 35°59′20.54″ N 14°22′28.41″ E, 15.IV.2020, with *M. capitatus*, leg. T. Cassar. GOZO: 2 exs., Ta’ Pinu, 36°3′32.435″ N 14°13′11.512″ E, 7.VI.2019, with *M. capitatus*, leg. A. Lapeva-Gjonova.

**Previous records**. [94,95].

**Global distribution**. Circum-Mediterranean; recorded in Europe from Spain eastwards to Greece, in North Africa from Morocco eastwards to Tunisia, and from Turkey [95].

**Local distribution and frequency**. A common, widespread species in Malta recorded from various locations.

**Ecology**. In the present study, this species has been found to occur in the nests of *Messor capitatus*. It appears that *C. formicaria* is an obligate nest-dweller of *Messor*, preferentially inhabiting the seed waste dumps of their host ants [98], indicating that this species is spermatophagous, mycetophagous, or both. It has been recorded as occurring with *Messor barbarous* Linnaeus, 1767 [98]. Other Mediterranean species of the genus *Cholovocera* also appear to be obligate nest-dwellers of *Messor* species [97].

***Merophysia formicaria* Lucas, 1852**

**Material examined**. None.

**Previous records**. [44,94,95].

**Global distribution**. Circum-Mediterranean [95].

**Local distribution and frequency**. This species does not appear to be common; only once is a specific location provided in which this species was collected—Manoel Island [44]; other works mentioned above simply state “Malta” [94,95]. See remarks below.

**Ecology**. All species of *Merophysia* occur “under stones in association with ants” in southern Italy, though any details on ecology appear to be unknown [96]. It has been stated that *Merophysia* species are found exclusively inside ant nests, in the company of ants, or in the abandoned tunnels of old ant nests, but an adequate explanation as to what the relationship between the beetles and ants truly is has not been found [99]. Unsurprisingly, little is known about the ecology of *M. formicaria* specifically. No ant hosts have been recorded for Maltese specimens. Presumably it is mycetophagous as are other myrmecophilous merophysiine endomychids [98].

**Remarks.** No material for this species has been collected since its first record [44], and the possibility that it was initially misidentified is not excluded. It should also be noted that *Merophysia lata* Kiesenwetter, 1872 has also been mentioned from Malta [94,95], but no material is actually presented in these works, nor is their original record cited. As a result, *M. lata* is here excluded from the myrmecophilous fauna of the Maltese Islands as there appears to be no basis for its record. However, *M. formicaria* is retained—albeit doubtfully due to the possibility of misidentification—as in its case material is indeed presented [44].

***Merophysia oblonga* Kiesenwetter, 1872**

**Material examined**. MALTA: 1 ex., Marsaxlokk, Xrobb l-Għaġin, 29.XI.1993, associated with ants, leg. D. Mifsud. GOZO: 1 ex., Dwejra, 10.V.1996, leg. D. Mifsud.

**Previous records**. [94,95].

**Global distribution**. Italy, Malta, Greece and Turkey [95].

**Local distribution and frequency**. This species does not appear to be exceedingly common or abundant wherever it does occur, however it has been collected from both Malta and Gozo suggesting that it may be a widespread species in the Maltese Islands.

**Ecology**. This species is often referred to as being “associated with ants” as is the case for all *Merophysia*; indeed, it has been collected inside ant nests with *Tetramorium caespitum*, *Aphaenogaster* and *Pheidole pallidula* elsewhere in Europe [97]. No specific ant hosts have been recorded for Maltese specimens, and precisely why *M. oblonga* occurs inside ant nests appears to be unknown. Presumably it is mycetophagous as are other myrmecophilous merophysiine endomychids [98].

#### 3.1.8. Coleoptera: Dermestidae Latreille, 1804

***Thorictus grandicollis* Germar, 1842**

**Material examined**. GOZO: 3 exs., Għadira Ta’ Sarraflu, 36°02′12.5” N 14°11′54.6” E, 5.VI.2019, with *Messor bouvieri*, leg. A. Lapeva-Gjonova.

**Previous records**. [44,100].

**Global distribution**. Most of Europe (Portugal eastwards to European Russia), Turkey, and North Africa (Morocco east to Egypt) [100].

**Local distribution and frequency**. Literature suggests that this is a frequent and widespread species [100]. It has been recorded from Rabat (Ta’ Baldu), Manoel Island, St. Thomas Bay, Ta’ Qali, Żejtun and Gћadira in Malta; in Gozo it has been recorded from Ramla Bay and Xwieni Bay [100], as well as Ta’ Sarraflu pond [present study].

**Ecology**. Unlike other species in this genus, *T. grandicollis* is not phoretic on ants, instead roaming around ant nests freely in search of plant detritus and other organic matter on which it feeds [101]. This species is considered a generalist myrmecophile, occurring inside ant nests such as those of *Messor*, *Pheidole* and *Camponotus*, displaying appeasement behaviour towards its hosts [102,103].

**Remarks**. There is little data about this species’ ecology in the Maltese Islands, apart from the single instance of its occurrence in the nest of *Messor bouvieri* Bondroit, 1918 as mentioned in the material presented above. Most records do not state in what situation the specimens were encountered, and thus it remains unclear if any have been taken from ant nests at all. The remark that multiple specimens were collected “under bark of *Eucalyptus*” [100] is in contradiction with the fact that *Thorictus* beetles are assumed to be “obligate myrmecophiles” [104]. Hence, the ecology of this species seems unclear.

#### 3.1.9. Zygentoma Börner, 1904: Lepismatidae Latreille, 1802

***Neoasterolepisma crassipes* (Escherich, 1905)**

**Material examined**. MALTA: 3♂♂ & 1♀, Qrendi, 35°49′27.98” N 14°26′54.76” E, 29.II.2020, with *Tapinoma simrothi* & *Messor capitatus*, leg. T. Cassar; 1♂ & 1 juv., Qrendi, 35°49′28.50″ N 14°26′45.94″ E, 16.V.2021, leg. T. Cassar; 1♀, same data, separate nest; 1♂, Misraћ Gћar Il-Kbir (l/o Siġġiewi), 35°51′12.11″ N 14°23′52.85″ E, 29.IX.2019, with *M. capitatus*, leg. T. Cassar; 3♂♂ & 1♀, Mellieћa, Gћar Tuta, 35°58′36.91″ N 14°19′42.13″ E, 13.X.2019, with *M. capitatus*, leg. T. Cassar; 1♂, Mellieћa, Rdum Tal-Madonna, 35°59′20.54″ N 14°22′28.41″ E, 15.IV.2020, with *M. capitatus*, leg. T. Cassar; 5♂♂ & 1♀, Pembroke, 35°55′58.42″ N 14°28′42.96″ E, 16.II.2020, with *Tapinoma simrothi* & *M. capitatus*, leg. T. Cassar; 1♂ & 1 juv., Msida, University of Malta Campus, 35°54′6.15″ N 14°29′4.47″ E, 5.V.2021, with *M. capitatus*, leg. T. Cassar. COMINO: 4♂♂, 36°00′45.6″ N 14°20′07.0″ E, 23.II.2020, with *Tapinoma simrothi* & *M. capitatus*; leg. T. Cassar. GOZO: 2♀♀, Ta’ Ċenċ (Sannat), 36° 1′15.63″ N 14°15′31.81″ E, 30.XII.2019, with *M. capitatus*, leg. T. Cassar; 3♂♂ & 1♀, same data, separate nest; 1♀, Rabat, 36°2′25.34″ N 14°14′39.48″ E, 28.II.2020, leg. B. Grech; 1♂ & 1♀, same data, 6.II.2020; Ramla Bay, 6.VI.2019, with *M. capitatus*, leg. A. Lapeva-Gjonova.

**Previous records**. [105,106].

**Global distribution**. Circum-Mediterranean [107].

**Local distribution and frequency**. A very common and widespread species recorded from various locations across all three of the main islands of the Maltese Archipelago (see material examined). This species has also been recorded from St Paul’s Islands and Gћajn Ħadid (Selmun) [105,106].

**Ecology**. From the present study, it appears that *N. crassipes* occurs exclusively with *Messor capitatus*, with multiple individuals occurring in a single nest, usually in the upper chambers just beneath a rock or other solid debris. *N. crassipes* has been previously described as a “*Messor* specialist” [108]. This silverfish species has been found to retain the chemical profile used as chemical mimicry of its host ant even after moulting, indicating that this taxon can endogenously synthesize *Messor* hydrocarbons [109]. *N. crassipes* has also been recorded from Malta as occurring “with *Messor*”; however this species has been found in the nest of *Pheidole pallidula* on one occasion [105,106]. The latter seems to contradict the assumption that *N. crassipes* is a *Messor* specialist, but consistent records of *N. crassipes* outside of *Messor* association seem to be nonexistent except for this one instance, and for the time being it is considered accidental.

***Neoasterolepisma wasmanni* (Moniez, 1894)**

**Material examined**. MALTA: 2♂♂ & 1♀, Imselliet Valley, l/o Żebbiegћ and Bidnija, 35°55′11.76″ N 14°23′46.60″ E, 10.V.2020, with *Camponotus barbaricus*, leg. T. Cassar; Marsaxlokk, 35°50′21.5″ N 14°32′53.5″ E, 20.XI.2017, with *Pheidole indica*, leg. A. Lapeva-Gjonova.

**Previous records**. [105,106].

**Global distribution**. Circum-Mediterranean [107].

**Local distribution and frequency**. This species appears to be less common than its congener *N. crassipes*; it has been recorded from distanced locations (Wied l-Imselliet & Marsaxlokk in the present study) which may indicate that it is, nevertheless, relatively widespread. It has been recorded this species from Wied Għammieq (Kalkara), Gћadira, San Luċjan (Marsaxlokk) and Ta’ Sarraflu pond in Gozo [105,106].

**Ecology**. In the present study, *N. wasmanni* was only ever encountered in the nests of *Camponotus barbaricus* and *Pheidole indica* Mayr, 1879. It has also been recorded from a nest of *Camponotus barbaricus* in Malta [106]. This seems to contradict the consideration of this species as a “*Messor* specialist” [108]. In fact, though multiple nests of *Messor* were sampled throughout Malta, Gozo and Comino in the present study, the only *Neoasterolepisma* species found to occur therein was *N. crassipes*. *N. wasmanni* has also been collected from a *Camponotus* nest, in association with *C. cruentatus* (Latreille, 1802) [109].

#### 3.1.10. Zygentoma: Nicoletiidae Escherich, 1905

***Proatelurina pseudolepisma* (Grassi, 1887)**

**Material examined**. MALTA: 1♂ & 1♀, Pembroke, 35°55′58.42″ N 14°28′42.96″ E, 14.II.2021, with *Tetramorium* (*semilaeve* group), leg. T. Cassar; 1♀, same locality, 2.IV.2021, with *Tapinoma simrothi*; 4♀♀ & 1 juv., same data but separate nest of *Tapinoma simrothi* & *Tetramorium* (*semilaeve* group); 1♀, Imselliet Valley, 35°55′4.84″ N 14°24′24.41″ E, 22.VII.2021, with *Linepithema humile*, leg. T. Cassar; Żurrieq, 35°49′20.3″ N 14°27′27.7″ E, 15.XI.2017, with *Pheidole pallidula*, leg. A. Lapeva-Gjonova; Wied Babu, 35°49′27.8″ N 14°27′36.4″ E, 20.XI.2017, with *P. pallidula*, leg. A. Lapeva-Gjonova; Marsaxlokk, 35°49′28.6″ N 14°33′23.4″ E, 2.VI.2019, with *T. simrothi*, leg. A. Lapeva-Gjonova.

**Previous records**. [105].

**Global distribution**. Circum-Mediterranean [107].

**Local distribution and frequency**. A frequent and apparently widespread species on the main island of Malta, recorded from Pembroke, Imselliet (Żebbiegћ–Bidnija), Żurrieq and Wied Babu in the present study. It has also been recorded from Mistra [105].

**Ecology**. Though a strict myrmecophile, *P. pseudolepisma* is certainly a generalist species when considering the wide range of potential ant hosts with which it occurs. In the present study, it was found to occur in nests of *Tetramorium* cf. *semilaeve*, *Tapinoma simrothi, Pheidole pallidula* and *Linepithema humile* (Mayr, 1868). All of these genera have previously been recorded as *P. pseudolepisma* hosts, as well as *Aphaenogaster, Bothriomyrmex, Camponotus, Cataglyphis, Formica, Lasius, Leptothorax, Messor* and *Plagiolepis* [110]. This species generally does not occur in very high numbers within the ant nests it inhabits, unlike *Neoasterolepisma*.

### 3.2. Collembola Lubbock, 1871

Paronellidae Börner, 1913

***Cyphoderus albinus* Nicolet, 1842**

**Material examined**. MALTA: 11 exs. observed, Chadwick Lakes, Rabat, 35°53′25.9″ N 14°22′57.5″ E, 16.XI.2022, with *Tetramorium* sp., obs. T. Cassar.

**Previous records**. [45,111].

**Global distribution**. Europe, North Africa and the Middle East [20].

**Figure 4:**
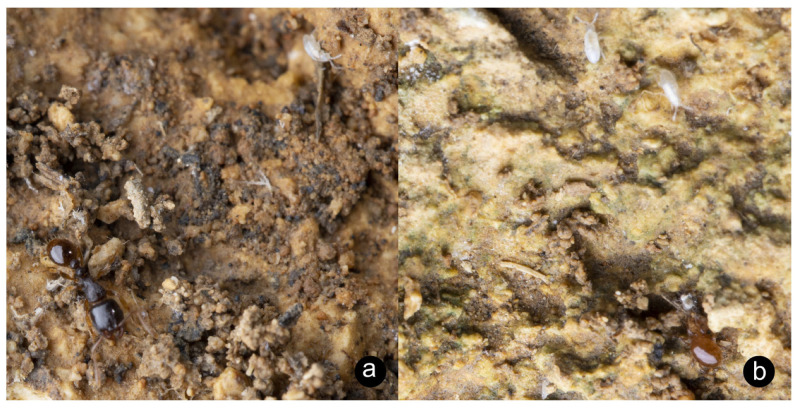
*Cyphoderus albinus* in an ants’ nest in Rabat; (**a**) one individual (top right) with *Tetramorium* worker (bottom left), Chadwick Lakes; (**b**) same nest as 4 (**a**), two *Cyphoderus albinus* (top) and a laelapid mite (bottom right).

**Local distribution and frequency**. Seven specimens were collected in “Floriano, under flower-pots” in July of 1925 by “Mr. Conte Guarano Gatto” [45]. Since then specimens have been observed in Rabat (Figure 4a,b). It is likely that this species is much more widespread but simply under-recorded.

**Ecology**. Though many springtails and other soil organisms occur in ant nests only as ‘accidental guests’, *Cyphoderus albinus* has been described as “panmyrmecophilous” [29] and an “obligate ant symbiont” [112]. It has been observed to occur in great numbers in the nests of *Camponotus, Lasius, Formica* and *Myrmica* [20]. Here, they feed on decaying plant matter and microorganisms, acting in turn as an important food source for myrmecophilous predators such as staphylinids and spiders [29].

### 3.3. Malacostraca Latreille, 1802

#### 3.3.1. Isopoda Latreille, 1817: Porcellionidae Brandt, 1831

***Porcellionides myrmecophilus* (Stein, 1859)**

**Material examined**. MALTA: 2♂♂ & 2♀♀, Qrendi, 35°49′27.98″ N 14°26′54.76″ E, 29.II.2020, with *Tapinoma simrothi* & *Messor capitatus*, leg. T. Cassar; 3♂♂ & 3♀♀, Mellieћa, Rdum Tal-Madonna, 35°59′20.54″ N 14°22′28.41″ E, 15.IV.2020, with *M. capitatus*, leg. T. Cassar; 1♂ & 2♀♀, Pembroke, 35°55′58.42″ N 14°28′42.96″ E, 16.II.2020, *T. simrothi* & *M. capitatus*, leg. T. Cassar; 1♀, Pembroke, 35°55′58.42″ N 14°28′42.96″ E, 2.IV.2021, with *M. capitatus*, leg. T. Cassar; 1♂ & 2♀♀, Msida, University of Malta Campus, 35°54′6.15″ N 14°29′4.47″ E, 5.V.2021, with *M. capitatus*, leg. E. Cutajar; COMINO: 1♂ & 2♀♀, 36°00′45.6″ N 14°20′07.0″ E, 23.II.2020, with *T. simrothi* & *M. capitatus*, leg. T. Cassar; GOZO: 4♂♂ & 4♀♀, Sannat, Ta’ Ċenċ, 36° 1′15.63″ N 14°15′31.81″ E, 30.XII.2019, with *M. capitatus*, leg. T. Cassar; same data,1 imm. in a different nest; 1♂, Rabat, 36°2′25.34″ N 14°14′39.48″ E, 28.II.2020, with *M. capitatus*, leg. B. Grech, same data, 2♂♂ in a different nest; same data, 1♀, 21.II.2020.

**Previous records**. [54,55,56].

**Global distribution**. Mediterranean—Greece (mainland, Crete, Dodecanese Islands, Cyclades, North Aegean Islands), Italy (including Sardinia and Sicily) and the Maltese Islands [56].

**Local distribution and frequency**. An exceptionally common and extremely widespread species, more or less evenly distributed over Malta, Gozo and Comino; it has also been recorded from St Paul’s Islands and Cominotto. It has previously been recorded in Malta from Wied Qirda, Wied il-Gћasel, Baћar iċ-Ċagћaq, il-Ballut tal-Imġiebaћ, Sa Maison (Pieta’), Gћar il-Friefet (Birżebbuġa), Gћar il-Kbir (Siġġiewi), Delimara; and in Gozo from Qolla l-Bajda (Żebbuġ), Gelmus & Ta’ Kuljat (Rabat) [56]. New locality records from the present study include Qrendi, Rdum Tal-Madonna (Mellieћa), Pembroke and Msida in Malta; and Ta’ Ċenċ (Sannat) in Gozo.

**Figure 5:**
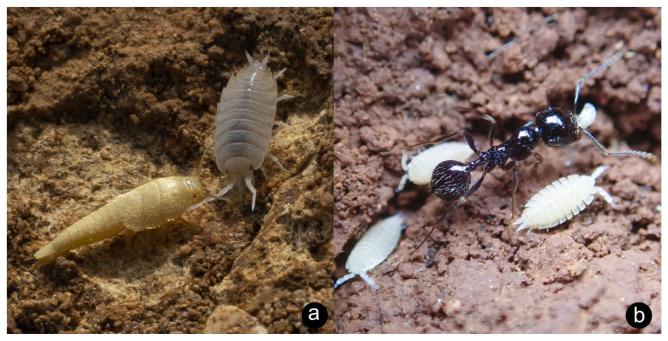
Myrmecophilous isopods and silverfish in Malta; (**a**) *Neoasterolepisma crassipes* (**left**) and *Porcellionides myrmecophilus* (**right**) in *Messor* nest, Rdum tal-Madonna; (**b**) *Platyarthrus* in *Messor* nest with worker carrying larva, Marfa.

**Ecology**. This species has been recorded as occurring in the nests of *Messor capitatus*, *Messor (?) structor* and *Tapinoma simrothi* [56], to which the observations of the present study closely match—multiple specimens were encountered almost exclusively in the nests of *M. capitatus* (Figure 5a). Like other myrmecophilous isopods, *P. myrmecophilus* feeds on decaying organic matter in the nest, possibly including stored seeds harvested by the ants themselves.

#### 3.3.2. Isopoda: Platyarthridae Verhoeff, 1949

***Platyarthrus aiasensis* Legrand, 1954**

**Material examined**. MALTA: 1♀, Pembroke, 35°55′58.42″ N 14°28′42.96″ E, 2.IV.2021, with *Tapinoma simrothi* & *Tetramorium* (*semilaeve* group), leg. T. Cassar; 1♀, Rabat, Chadwick Lakes, 35°53′52.10″ N 14°23′50.89″ E, 25.VII.2021, with *Lasius lasioides*, leg. T. Cassar.

**Previous records**. [54,55,56].

**Global distribution**. Western Mediterranean, North & Central America, South Africa [113].

**Local distribution and frequency**. This species appears to be fairly widespread, having been recorded from the following locations in Malta: Wied il-Gћasel (Mosta), Mistra Bay, Gћar Lapsi (Siġġiewi), Baћar iċ-Ċagћaq, Bengћisa, Gћajn Ħadid (Selmun), Chadwick Lakes (Rabat), Wied Inċita (Attard), Wied Magћlaq (Qrendi), il-Ballut ta’ Marsaxlokk, Gћadira s-Safra, Gћallis (Naxxar), Pembroke and Wied Babu (Żurrieq); in Gozo it has been recorded from Ta’ Ċenċ (Sannat), Ramla Valley, Gћasri Valley, Ġnien Imrik (Xagћra), Xlendi Valley, Gelmus (Rabat), Qolla l-Bajda (Żebbuġ); it has also been recorded from St Paul’s Islands, Cominotto, Filfla and Fungus Rock [54,56].

**Ecology**. This species has a wide range of ant host species in the Maltese Islands, namely *Lepisiota frauenfeldi velox* (Baroni Urbani, 1968)*, Plagiolepis pygmaea, Pheidole pallidula*, an unidentified *Solenopsis* species, *Tapinoma simrothi, Messor capitatus* and *M.* (?) *structor* [56]. The present study adds *Tetramorium* and *Lasius lasioides* (Emery, 1869) to this list of local ant hosts. *P. aiasensis* may form parthenogenetic populations, though this does not appear to be the case locally [113].

***Platyarthrus caudatus* Aubert & Dollfus, 1890**

**Material examined**. MALTA: 5 exs., Qrendi, 35°49′27.98″ N 14°26′54.76″ E, 29.II.2020, with *Tapinoma simrothi* & *Messor capitatus*, leg. T. Cassar; 1♂ & 1♀, Valletta, 35°53′42.57″ N 14°30′38.54″ E, 9.V.2020, with *Tetramorium* (*caespitum* group), leg. T. Cassar; 1♀, Imselliet Valley, 35°55′4.84″ N 14°24′24.41″ E, 22.VII.2021, with *Linepithema humile*, leg. T. Cassar; 2♂♂, Mosta, Tal-Wej, 35°54′59.75″ N 14°26′2.98″ E, 20.X.2019, with *M. capitatus*, leg. T. Cassar; 1 ex. Rabat, Chadwick Lakes, 35°53′52.10″ N 14°23′50.89″ E 25.VII.2021, with *Camponotus barbaricus*, leg. T. Cassar.

**Previous records**. [54,55,56].

**Global distribution**. Western Euro-Mediterranean; from mainland Spain eastwards to Italy, including various islands such as the Balearic Is., Corsica, Sardinia, Sicily and the Maltese archipelago [56].

**Local distribution and frequency**. Appears to be a common and fairly widespread species, albeit under-recorded from Southeastern Malta. It has previously been recorded from the following locations in Malta: Chadwick Lakes (Rabat), Mistra Bay, Wied Inċita (Attard), Gћadira, Paradise Bay, St Maria Estate & Imġiebaћ (Mellieћa), Wied il-Luq & Buskett (Siġġiewi), Salini saltmarsh, il-Magћluq ta’ Marsaskala, Fawwara, Gћajn Ħadid (Selmun), Baћar iċ-Ċagћaq and Gћallis (Naxxar); from Gozo it has been recorded from Xlendi Valley, Wied Ħanżira, Sara Valley and Ta’ Kuljat (Rabat); as well as from Comino, Cominotto and Filfla [54,56]. The present study adds Qrendi, Valletta, Wied l-Imselliet and Tal-Wej (Mosta) to its local distribution, confirming its widespread nature in Malta.

**Ecology**. Myrmecophilous but not host-specific. It is known to occur in the nests of *Plagiolepis pygmaea, Pheidole pallidula, Solenopsis, Tapinoma simrothi* and *Camponotus lateralis* (Olivier, 1792) [56]. It also occurs with *Messor capitatus, Tetramorium, Linepithema humile* and *Camponotus barbaricus* [present study].

***Platyarthrus schoblii* Budde-Lund, 1885**

**Material examined**. MALTA: 10 exs., Qrendi, 35°49′27.98″ N 14°26′54.76″ E, 29.II.2020, with *Messor capitatus*, leg. T. Cassar; 2♂♂ & 3♀♀, Pembroke, 35°55′58.42″ N 14°28′42.96″ E, 16.II.2020, with *Tapinoma simrothi* & *M. capitatus*; 2 exs., Mellieћa, Gћar Tuta, 35°58′36.91″ N 14°19′42.13″ E, 13.X.2019, with *M. capitatus*; COMINO: 8 exs., 36°00′45.6″ N 14°20′07.0″ E, 23.II.2020, with *M. capitatus*, leg. T. Cassar; same data, 1♂ & 5♀♀ with *T. simrothi* & *M. capitatus*; 1♂ & 5♀♀, 36° 0′50.53″ N 14°19′48.24″ E, 10.IV.2021, with *T. simrothi* & *M. capitatus*; GOZO: 32 exs., Gozo, Sannat, Ta’ Ċenċ, 36° 1′15.63″ N 14°15′31.81″ E, 4.I.2021, with *M. capitatus*, leg. T. Cassar; same data, 12 exs., 30.XII.2019.

**Previous records**. [54,55,56].

**Global distribution**. Holomediterranean, introduced to Hungary [114].

**Local distribution and frequency**. Appears to be a fairly widespread and common species. Recorded from Mistra Bay, Paradise Bay, il-Magћluq ta’ Marsaskala, Fawwara, Gћallis (Naxxar) and Marfa [54,56]. Specimens from Pembroke, Gћar Tuta (Mellieћa) and Qrendi in the present study add to its known distribution in Malta, whereas specimens from Comino and Gozo appear to be the first records for these islands.

**Ecology**. This species has been recorded previously as occurring with *Tapinoma simrothi* [56]. Multiple specimens were also encountered in *Messor capitatus* nests in the present study, and various *Lasius* species and *Tetramorium caespitum* have been recorded as hosts for *P. schoblii* elsewhere in Europe [114].

**Remarks**. Two subspecies have been recorded from the Maltese Islands; *P. s. intermedius* and *P. s. esterelanus* [56]. However, the taxonomic status of many subspecies of *P. schoblii* is unclear; furthermore many taxa exist in a species complex whose systematics remains unresolved [115]. In the present work, some individuals belonged to an unidentified subspecies of *P. schoblii* in which the dorsal sculpturing and male pleopods differed from those in any known subspecies (Garcia Socias, *pers. comm.*, 2021). Taxonomic resolution is outside of the scope of the present work; for the time being all individuals shall be listed solely at the specific level.

### 3.4. Arachnida Lamarck, 1801

#### 3.4.1. Mesostigmata G. Canestrini, 1891: Laelapidae Berlese, 1892

***Gymnolaelaps messor* Joharchi, Halliday, Saboori & Kamali, 2011**

**Material examined**. MALTA: Mosta, Tal-Wej, 35°54′59.75″ N 14°26′2.98″ E, 20.X.2019, with *Tapinoma simrothi* & *Messor capitatus*, leg. T. Cassar. COMINO: 36°00′45.6″ N 14°20′07.0″ E, 23.II.2020, with *M. capitatus*, leg. T. Cassar.

**Previous records**. New record for the Maltese Islands.

**Global distribution**. Iran [116] and Malta.

**Local distribution and frequency**. Though its true distribution remains to be fully understood as the above material represent the first records of this species in Malta, *G. messor* appears to be relatively widespread in the Maltese archipelago as it has been collected from mainland Malta as well as the island of Comino.

**Ecology**. The ecological role of myrmecophilous laelapid mites is poorly known. Most references to their ecology in the literature state simply that the mites are “closely associated” with a given host ant, found exclusively inside ant nests, but no information is provided about what the mites feed on or their relationship with the ants [117]. Many myrmecophilous laelapid mites are, however, likely to be predators of other nest-associated invertebrates, including other mites, and may either be commensals inhabiting ant nests or exist in a mutually beneficial relationship with their hosts [118]. In Iran, from which the type material of this species was collected, *G. messor* was also found in association with an unidentified *Messor* ant species [116]. Local investigations appear to confirm its affinity for *Messor*, with multiple individuals being collected inside the nests of *Messor capitatus* in karstic phrygana. Otherwise, little is known about the finer details of this species’ ecology.

***Gymnolaelaps myrmecophilus* (Berlese, 1892)**

**Material examined**. MALTA: 1♀, Rabat, Chadwick Lakes, 35°53′52.10″ N 14°23′50.89″ E, 25.VII.2021, with *Lasius lasioides*, leg. T. Cassar; 5 exs., same locality, 3.X.2021.

**Remarks**. The above-mentioned material corresponds to a taxon originally described as *Hypoaspis myrmecophila* var. *longisetosa* [119]. It is thus considered as a distinct variety of *Gymnolaelaps myrmecophilus* (=*Hypoaspis myrmecophila*) (Berlese, 1892). *Gymnolaelaps myrmecophilus* is a widespread myrmecophilous species present in the Canary Islands, most of Europe and Iran, occurring in nests of *Tetramorium* and *Formica* [120,121,122]. The variety “*longisetosa*” was only collected twice—once in 1899 and once in 1900—from ants’ nests in San Remo (Italy) and this material was used to describe this variety (Joharchi, *pers. comm*, 2022).

According to the International Code of Zoological Nomenclature, taxa described as varieties (“var.”) of species prior to 1961 should be considered as subspecies, as infrasubspecific ranks are no longer recognized [123]. Thus, the Maltese material should more correctly be referred to as *Gymnolaelaps myrmecophilus longisetosus* (Oudemans, 1902).

However, in the present work, the taxon is being cited at species level for the following reasons: (i) since its original description in 1902, the var. “*longisetosa*” was never again collected or mentioned in the scientific literature; (ii) there is no recent taxonomic treatment of this mesostigmatan; (iii) the “var. *longisetosa*” was inadequately described, its original description consisting only of a single sentence in Dutch, as follows: “Similar to the type [of *Hypoaspis myrmecophila*], except that all body hairs are long and fine”. Nevertheless, material utilized by Oudemans to describe it has been tracked down to the Naturalis Biodiversity Centre in Leiden, and the specimens—though not in perfect condition—clearly belong to the same taxon as do specimens collected in the present study from Malta (Joharchi, *pers. comm.*, 2022).

***Pogonolaelaps canestrinii* (Berlese, 1903)**

**Material examined**. MALTA: Qrendi, 35°49′27.98″ N 14°26′54.76″ E, 29.II.2020, with *Tapinoma simrothi* & *Messor capitatus*, leg. T. Cassar; Rabat, Chadwick Lakes, 35°53′42.04″ N 14°23′37.22″ E, 25.VII.2021, with *Pheidole pallidula*, leg. T. Cassar.

**Previous records**. New record for the Maltese Islands.

**Global distribution**. Italy, Malta and Iran [124].

**Local distribution and frequency**. Though its true distribution remains to be fully understood as the above material represents the first record of this species in Malta, *P. canestrinii* appears to be possibly widespread in Malta for a number of reasons: (i) it has been collected from two locations which are relatively far apart; (ii) it has been collected from two locations which differ greatly in habitat—a windswept coastal garigue plateau and a *Eucalyptus* stand bordering a freshwater stream; and (iii) it has been collected within the nests of different species of ants with quite distinct ecologies.

**Ecology**. Refer to general laelapid ecology mentioned for *Gymnolaelaps messor*.

#### 3.4.2. Araneae Clerck, 1757: Phrurolithidae Banks, 1892

***Phrurolithus* sp.**

**Material examined**. MALTA: 1 juvenile, Rabat, Chadwick Lakes, 35°53′42.04″ N 14°23′37.22″ E, 25.VII.2021, with *Pheidole pallidula*, leg. T. Cassar.

**Previous records**. New family record for the Maltese Islands.

**Global distribution**. The genus *Phrurolithus* C.L. Koch, 1839 is the most widespread of its family, with some 60 species distributed throughout North and Central America, Europe and Asia. Ten species can be found in the Mediterranean, with the following four species inhabiting southern Europe and being possible candidates for the identity of the specimen collected in this study: *Phrurolithus festivus* (C.L. Koch, 1835), *P. minimus* C.L. Koch, 1839*, P. nigrinus* (Simon, 1878) and *P. szilyi* Herman, 1879 [125].

**Local distribution and frequency**. So far, this species has only been collected at Chadwick Lakes (Rabat), though its small size and low population density within ant nests may represent challenges to sampling efforts, obscuring its true local distribution.

**Ecology**. Most spiders of the family Phrurolithidae have been recorded living in association with ants, preying on formicid workers as they gather resources for the nest, or the insects which are flushed out by foraging ant trails [126]. A few, however, have been collected within the ant nest structure itself. *Phrurolithus festivus* and *P. minimus* have been observed both inside and outside ant nests, indicating that they are only occasional nest-inhabitants; *Otacilia komurai* (Yaginuma, 1952) and *Phruronellus formica* (Banks, 1895) are always observed inside the nest structure of ants and most likely represent obligate myrmecophiles [127,128,129,130]. The ecology of the spider collected in the present study is difficult to ascertain; by being present as a juvenile within the internal chambers of an ant’s nest it may be considered an intranidal myrmecophile. *Phrurolithus* spiders escape hostile treatment by their hosts through imperfect myrmecomorphy, in that their colouration and body shape only somewhat resemble that of their prey-hosts, and therefore the spiders must move rapidly in order to escape detection as continuous movement does not allow ants to distinguish the spiders from fellow formicids [131]. So far, *Phrurolithus* species have been found in association with *Myrmica, Lasius, Tapinoma* and *Formica* [22] and the association with *Pheidole* reported in the present study appears to be new.

**Remarks**. Though the habitus of the collected specimen can be assigned with certainty to the genus *Phrurolithus*, a species-level identification cannot be made as it is still too immature and specific diagnostic morphological characters are not well-developed (Marusik, *pers. comm*., 2021).

## 4. Discussion

### 4.1. Trends in Maltese Ant-Myrmecophile Associations

It is noteworthy that the greatest diversity of myrmecophilous arthropods encountered in the present study was that occurring in the nests of *Messor* species. Indeed, of the thirty species here regarded as true myrmecophiles, fourteen (c. 47%) are hosted by *Messor* (Table A1). A number of reasons could be responsible for this. *Messor* is represented by some very common species in the Maltese Islands, particularly *M. capitatus* which appears to be virtually euryecious and exceptionally widespread in the Maltese archipelago (*pers. obs.* T. Cassar). This, paired with the fact that *Messor* tends to form extensive, conspicuous nests under large rocks with visible nest-clearings, may have resulted in a sampling bias in *Messor*’s favour. Sampling comparatively more nests of one ant genus may certainly yield more myrmecophile diversity, not necessarily because that genus is an inherently better host, but simply due to a higher chance of encountering ‘new’ myrmecophiles.

However, an ecological explanation for exceptional intranidal myrmecophile diversity of *Messor* nests also exists. Harvester ants such as *Messor* construct extensive, structurally complex nests which are stocked with large quantities of seeds—*Messor* has been regarded as an ecosystem engineer for both surface vegetation and soil biota [132]. By harvesting seeds and other plant material, *Messor* allows for various trophic relationships to occur in a single nest; granivorous myrmecophiles may feed on seed stores; detrivorous and mycetophagous myrmecophiles on decaying plant matter; predatory myrmecophiles may prey on the ants themselves or other myrmecophilous arthropods [132,133]. The complexity of *Messor* nests and the digging of multiple chambers and tunnels is also favourable for soil microorganisms and small arthropods which aid nutrient cycling and the development of mycorrhizae, in turn increasing biomass and biological activity inside the ant nest [134,135]. Thus, the nests of *Messor* and other harvester ant genera tend to be biodiversity hotspots, with a wide range of arthropod taxa becoming adapted for life in their nests, some even evolving the ability to mimic *Messor* nest odour [98].

In fact, of the thirty obligate myrmecophiles mentioned in the present work, only five species are known with relative certainty to occur with only one ant genus, and four of those five occur strictly with *Messor*: the mite *Gymnolaelaps messor*, the silverfish *Neoasterolepisma crassipes* and two endomychid beetles of the genus *Cholovocera*, all of which have only ever been recorded with *Messor*, at times being described as ‘*Messor* specialists’ [98,108,116]. This appears to correspond with broader ant-association studies in which *Messor* was found to have among the highest proportions of unique ant symbiotes of any ant genus across Europe, and though other genera such as *Myrmica, Formica, Camponotus* and *Lasius* have higher total numbers of ant symbiotes (both unique and shared), this may simply be a result of having more species described in comparison to *Messor* [136]. The only other myrmecophile found to be restricted to one ant genus in the Maltese Islands was *Italochrysa italica*, whose larvae are associated with solely *Crematogaster* nests [89]. All other myrmecophilous species recorded in the present work are associated with at least two ant genera; the opposite side of the myrmecophilous spectrum being the so-called ‘panmyrmecophilous’ species with incredibly broad host ranges, most notably *Cyphoderus albinus*, various *Platyarthrus* species and *Proatelurina pseudolepisma,* the latter of which has at least fourteen known ant-hosts.

**Figure 6:**
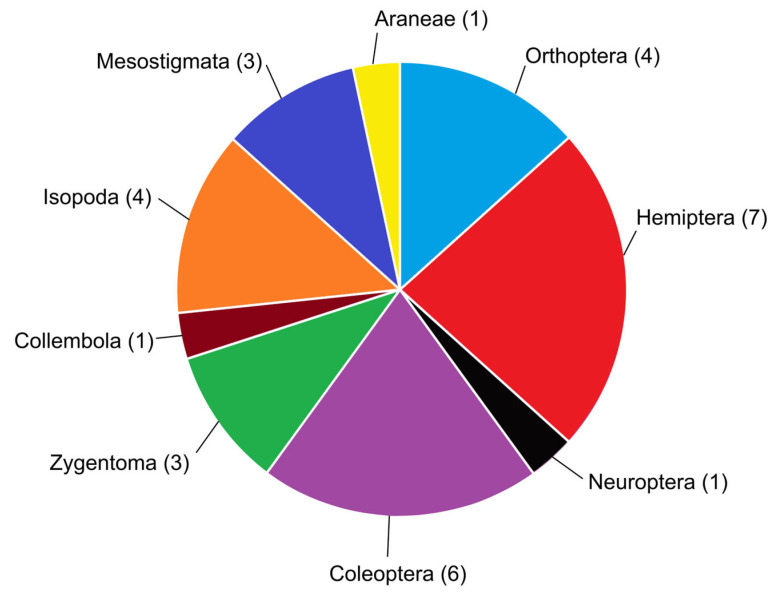
Pie chart showing representation of each arthropod group found to be intranidal myrmecophiles in the Maltese Islands; the number in parentheses denotes the number of intranidal myrmecophilous species from each group which occur in the archipelago.

The arthropod order with the most myrmecophilous representatives in Malta was found to be Hemiptera (Figure 6). This is perhaps to be expected as all species are trophobionts; the phytophagous, honeydew-excreting nature of many hemipterans lends itself well to striking up an intranidal partnership with ants. The second most diverse order with respect to intranidal myrmecophily in the Maltese Islands is Coleoptera, doubtless as a result of the order’s diversity as a whole. Interesting to note is the stark absence of the order Diptera, which tends to have a few myrmecophilous species in most nearby territories of the Mediterranean, such as species of *Microdon* Meigen, 1803, none of which occur in the Maltese Islands. Hemiptera’s lead in this study may only be temporary; further studies on Maltese myrmecophiles may reveal many more species and some arthropod groups are expected to be more well-represented than presently known—especially Coleoptera, with further sampling efforts to investigate myrmecophilous staphylinids, for example.

### 4.2. Occasional and ‘Accidental’ Ant Guests

Twelve arthropod species were encountered in the present study which, though occurring in ant nests, cannot be considered obligate myrmecophiles—three pseudoscorpions, one mite, three isopods and five beetles (Appendix A, Table A1). Their presence inside formicid nest structures is purely accidental or only occasional, and indeed none of such arthropods are in possession of adaptations to a myrmecophilous lifestyle, and can be found living in complete isolation from ant nests.

This is certainly the case for the pseudoscorpions *Microcreagrina hispanica* (Ellingsen, 1910), *Hysterochelifer tuberculatus* (Lucas, 1849) and *Pselaphochernes lacertosus* (L. Koch, 1873). Pseudoscorpions occurring in ant nests may be true myrmecophiles, in which all life stages occur in association with ants; others occur regularly in ant nests but not exclusively; while other species occur in ant nests only occasionally due to their soil-inhabiting nature [137]. In all cases, pseudoscorpions prey on small arthropods, often hunting smaller myrmecophilous invertebrates such as mites and springtails, and their formicid hosts pay them little attention [24,138]. No exclusively myrmecophilic genera, such as *Myrmochernes* Tullgren, 1907, were encountered in the present study. The occurrence of multiple developmental stages of a particular pseudoscorpion species in an ant nest is usually indicative of myrmecophily [22,139]. Nevertheless, the presence of both tritonymphs and adults of *Microcreagrina hispanica* and *Pselaphochernes lacertosus* with *Messor* and *Tetramorium* in the present study is here considered to be accidental—elsewhere in Europe, both of these species have been found living in soil, humus and compost heaps with no association whatsoever with ants or their nest structure [140,141]. *Hysterochelifer tuberculatus,* of which multiple adults were collected with *Messor* and *Lasius* in the present work, should also be considered as an ‘accidental guest’ in ant nests, as it usually occurs under tree bark, stones and phoretically on the bodies of wasps, with no association with ants or their nest structures [142,143]. None of the pseudoscorpions encountered were present in large numbers either, which also indicates their non-myrmecophilous nature [23].

Similarly, the occurrence of an unidentified rake-legged mite of the genus *Microcaeculus* Franz, 1952 in a *Camponotus* nest is also not considered to be a case of obligate myrmecophily. Rake-legged mites tend to occur under stones in arid environments where they prey on small arthropods such as springtails [144]. For this reason, they may occasionally occur in low numbers in ant nests which provide a bounteous supply of small arthropod prey [145]. Incidentally, this is the first record of the family Caeculidae in the Maltese Islands.

Three isopod species namely *Platyarthrus lerinensis* Vandel, 1957, *Leptotrichus naupliensis* (Verhoeff, 1901) and *L. panzeri* (Audouin, 1826) are here also not considered to be obligate myrmecophiles. *P. lerinensis* has been deemed to be ‘occasionally’ myrmecophilic, sometimes occurring with *Pheidole pallidula* but often found living outside of any association with ants [56]. Indeed, no specimens of *P. lerinensis* were encountered in the present study. *Leptotrichus* species, which were indeed collected in ants’ nests in the present study, are endogean detritivorous isopods which commonly occur in soil with or without ants [56].

A number of small beetles were also collected from ant nests. Of these, the histerid *Kissister minimus* (Laporte, 1840), an unidentified staphylinid belonging to either *Micranops* Cameron, 1913 or *Scopaeus* Erichson, 1839 and an unidentified scydmaenine are all predatory coleopterans which do not exclusively occur in ant nests (Lackner, Assing, Mifsud, *pers. comm*., 2021). These beetles are known to feed on small arthropods and their presence in ant nests may simply represent an occasional foray in order to prey on mites and springtails which occur in ant nests. This is especially true for scydmaenines, which are known to preferentially feed on oribatid and mesostigmatan mites—the latter of which tend to be abundant in some ant nests [146]. Though some scydmaenines are indeed myrmecophilic, those which are tend to show overt morphological adaptations to such a lifestyle which are lacking in the specimens observed in the present study [147]. Without a more precise identification, however, little can be said about the true ecology of this species. The presence of the endemic tenebrionid *Stenosis melitana* Reitter, 1894 under stones covering ant nests appears to be more enigmatic. It is possible that *Stenosis* species are simply found under stones as they overwinter and that no association exists between them and ants which happen to nest under stones; a previous study on Maltese tenebrionids revealed that adults of this genus can often be found sheltering under bark during the winter months [92]. In fact, *Stenosis* specimens were never retrieved from within the ant nest chambers in the soil, instead being found resting on the underside of stones lying on top of ant nests.

A large number of Maltese specimens of an unidentified anamorphid of the genus *Symbiotes* Redtenbacher, 1849 have also been examined in the present study. Species of this genus are sometimes collected in ant nests and the generic name most likely stems from an assumed myrmecophilous nature [148]. However, *Symbiotes* species have also been collected from rotting tree-holes, birds’ nests and decaying leaf litter, suggesting that they are generalist fungivores which occur wherever fungal hyphae and spores are available for consumption [95,149]. Indeed, collection specimens examined in the present study were labelled as being collected “under the bark of *Acacia* trees”, “under the bark of *Ceratonia siliqua*” and “under pigeon dung”. Thus, *Symbiotes* is here not considered to be an obligate myrmecophile; rather an opportunistic fungivore.

### 4.3. Potential Local Myrmecophiles with Unknown Associations

The syrphid fly genus *Chrysotoxum* is thought to have myrmecophilous larvae. Larvae of *C. festivum* (L.) have been collected from nests of *Lasius,* and *C. bicinctum* (L.) has been observed ovipositing in the vicinity of *Lasius* nests [150,151]. It is possible that *Chrysotoxum* larvae feed on root aphids found in ant nests—*C. bicinctum* has been observed to prey on aphids under controlled conditions [151]. The only species of *Chrysotoxum* which occurs in Malta is *C. intermedium* Meigen, 1822 [152]. Though habitat preference and flight phenology for adults of this species in the Mediterranean have been studied [153], the larva of *C. intermedium* has never been collected, described or reared. No syrphid larvae were encountered in the present work within ant nests, and until the biology of *C. intermedium* is elucidated, it is regarded as a potential local myrmecophile awaiting confirmation—based on the known life history of congeners elsewhere in Europe.

Another potentially myrmecophilic insect which may occur locally is *Reptalus panzeri* (Löw, 1883). Observations have been made of *R. panzeri* occurring in the nests of *Aphaenogaster subterranea* (Latreille, 1798), though the relationship between the cixiid and its ant hosts remains unknown—assumed to be trophobiotic [9]. The identity of the *Reptalus* species occurring in Malta is still uncertain, though it has been tentatively assigned to *R.* cf. *panzeri* [47]. Three taxa of the *A. subterranea* group also occur in Malta, though they have not been definitively identified to species level either [58]. The possibility that *R. panzeri* is a locally occurring myrmecophilic cixiid is not excluded; confirmation would require full identification of the cixiid and possible host taxa which occur in Malta, as well as improved knowledge of the association between *R. panzeri* and *A. subterranea* in general.

### 4.4. Future Avenues for Maltese Myrmecophile Research

The present work does not represent a complete understanding of the ecology and diversity of myrmecophilous arthropods in the Maltese Islands. The archipelago hosts at least seventy species of ant [58]; nests belonging to only thirteen species were sampled in the present work. Undoubtedly, more rigorous efforts to examine the nests of more ant species will reveal additional myrmecophilous taxa and new associations. Of course, some ant species are rare in the Maltese Islands and finding their nests may require more effort than others. Other ant nests may be difficult to sample not because the species are rare, but because of the nature of their nests. For example, no nests of *Crematogaster scutellaris* were examined in the present study despite the authors locating them on several occasions, as the nests were constructed in the heart of tree trunks. Breaking open the tree trunk would have resulted in the complete destruction of the ant nest, and in some cases caused damage to legally protected tree species, and thus their nests were left untouched. Other ant genera construct hypogaeic nests deep underground and thus are not easily detected; some ants are exceptionally small and rarely collected (e.g., *Leptanilla*), and others reside in very small nests which easily escape detection (e.g., *Temnothorax*) [154,155]. Searching for the nests of cryptic, hypogaeic-nesting ants may reveal particularly interesting associations for lesser-known myrmecophiles.

The hymenopteran parastoids of ants in the Maltese Islands are also unknown; as intranidal ant-associated arthropods, they too must be considered myrmecophiles. A number of eucharitid wasps occur in the Mediterranean which attack the immature stages of cocooning ants such as *Camponotus*, including *Stilbula cyniformis* (Rossi, 1792) and *Eucharis punctata* Foerster, 1859 [15]. Rearing ant cocoons under observation may yield previously unrecorded hymenopteran parasitoids; so far no eucharitids have been recorded from Malta despite the presence of suitable hosts [156]. A number of ant-associated diapriids also occur in the Holarctic, such as *Lepidopria pedestris* Kieffer, 1916, *Plagiopria passerai* Huggert & Masner, 1983 and *Solenopsia imitatrix* Wasmann 1899, but no myrmecophilous diapriids have been recorded from the Maltese Islands so far [157,158].

Finally, a biogeographic analysis of Maltese myrmecophiles supported by molecular data may reveal interesting relationships between the fauna of the archipelago and other Mediterranean territories. For example, the occurrence of the wingless cricket *Myrmecophilus baronii* in Malta, Pantelleria and Tunisia raises interesting questions, as “*there is no completely unequivocal evidence of direct…connections*” between the Maltese Islands and mainland Africa since the Messinian Salinity Crisis some 5.3 million years ago [159]. Mitochondrial haplotypes and other molecular analyses may elucidate the relationship between these isolated Mediterranean populations with no apparent natural means of dispersal—perhaps this species was transported passively alongside host ants by humans, as was *Myrmecophilus quadrispinus*? Other unexpected myrmecophile distributions may be explained simply by gaps in faunistic knowledge, such as *Gymnolaelaps messor* which has only been recorded from Iran and Malta—this is almost certainly due to a lack of in-depth acarological study in many territories, and future works may reveal that this species actually occurs in many more countries.

Certainly, such an unexpected diversity of arthropod myrmecophiles in the relatively small and habitat-poor islands of the Maltese archipelago warrants immediate conservation action. Some species, such as *Myrmecophilus baronii* and *Trama baronii*, have not been recorded locally in many decades despite concerted efforts to locate them, indicating low population densities susceptible not only to stochastic events but, more concerningly, the main driver of extinction in the Anthropocene—human activity. The Maltese Islands have been undergoing rapid urbanization, with reclamation of natural areas for construction of human habitation, quarrying and dumping having a direct and drastic impact on local species, especially stenotopic taxa [160].

## 5. Conclusions

The intranidal myrmecophilous arthropod fauna of the Maltese Islands is found to be relatively rich, with a diverse array of arthropods from nine orders existing in close association with various ant genera in the archipelago. Though most of these species were locally recorded in the literature prior to the present work, their biological associations were scarcely elaborated. Moreover, among the species recorded in the present work, a number of taxa are recorded for the first time from the Maltese Islands and Europe. This, paired with the fact that nests from only about one fifth of the known ant diversity in Malta were sampled, indicates that much more is left to be revealed in the field of Maltese myrmecophilology. The nests of *Messor* harvester ants have proved to be considerable repositories of myrmecophile diversity.

## Data Availability

Data is contained within the manuscript.

## Appendix A

**Table A1:** Maltese intranidal arthropod myrmecophiles associated with each of the ant genera whose nests were sampled in the present study. Taxa listed in **black** were either observed directly in association with the ant genera in the present study, or have been recorded with such genera in Malta in previous works. Taxa listed in **blue** have not been observed directly in association with such ant genera in Malta, but likely do occur in their nests locally as they have been recorded in association with such ant genera elsewhere in Europe or the Mediterranean region, and they are present in the Maltese Islands. These taxa are indicated after the “+” symbol in the rightmost column showing total number of ant-myrmecophile associations in the Maltese Islands.

Sampled Ant Genus	Intranidal Myrmecophile Associates Recorded from the Maltese Islands	Total Locally Recorded Associations + Probable Additional Associations
*Messor*	*Myrmecophilus ochraceus, Myrmecophilus baronii, Cholovocera fleischeri, Cholovocera formicaria, Thorictus grandicollis, Neoasterolepisma crassipes, Porcellionides myrmecophilus, Platyarthrus caudatus, Platyarthrus schoblii, Gymnolaelaps messor, Pogonolaelaps canestrinii,* *Proatelurina pseudolepisma, Neoasterolepisma wasmanni, Platyarthrus aiasensis*	11 + 3
*Pheidole*	*Myrmecophilus ochraceus, Rectinasus buxtoni, Neoasterolepisma wasmanni, Proatelurina pseudolepisma, Pogonolaelaps canestrinii, Phrurolithus* sp., *Merophysia oblonga, Thorictus grandicollis, Platyarthrus aiasensis, Platyarthrus caudatus*	6 + 4
*Tetramorium*	*Myrmecophilus ochraceus, Proatelurina pseudolepisma, Cyphoderus albinus, Platyarthrus aiasensis, Platyarthrus caudatus,* *Tettigometra atra, Tettigometra laeta, Merophysia oblonga, Platyarthrus schoblii*	5 + 4
*Camponotus*	*Myrmecophilus baronii, Trama baronii, Neoasterolepisma wasmanni, Platyarthrus caudatus,* *Myrmecophilus fuscus, Tettigometra atra, Thorictus grandicollis, Proatelurina pseudolepisma, Cyphoderus albinus*	4 + 5
*Lasius*	*Platyarthrus aiasensis, Gymnolaelaps* cf. *myrmecophilus, Myrmecophilus fuscus, Tettigometra atra, Tettigometra laeta, Proatelurina pseudolepisma, Cyphoderus albinus, Platyarthrus schoblii*	2 + 6
*Tapinoma*	*Tettigometra impressifrons, Lacombia dactyloni, Proatelurina pseudolepisma, Porcellionides myrmecophilus, Platyarthrus aiasensis, Platyarthrus caudatus, Platyarthus schoblii*	7 + 0
*Plagiolepis*	*Smynturodes betae,* *Proatelurina pseudolepisma, Platyarthrus aiasensis, Platyarthrus caudatus*	1 + 3
*Linepithema*	*Proatelurina pseudolepisma*, *Platyarthrus caudatus*	2 + 0
*Crematogaster*	*Italochrysa italica, Myrmecophilus fuscus*	0 + 2

**Table A2:** Taxa whose presence in ant nests is considered only occasional or purely accidental, or whose myrmecophilous status is otherwise ambiguous ^1^.

Taxon	Material Examined
Arachnida: Pseudoscorpiones	
*Microcreagrina hispanica* (Ellingsen, 1910)	Malta: 1 tritonymph, Qrendi, 35°49′27.98″ N 14°26′54.76″ E, 29.II.2020, with *Messor capitatus*, leg. T. Cassar.
*Hysterochelifer tuberculatus* (Lucas, 1849)	Malta: 1♂ & 1♀, Qrendi, 35°49′28.50″ N 14°26′45.94″ E, 16.V.2021, with *Messor capitatus,* leg. T. Cassar; 1♂, Mġarr, Lippija Tower, 35°55′19.85″ N 14°20′44.31″ E, 11.IV.2021, with *Messor capitatus,* leg. T. Cassar; 1♂, Rabat, Chadwick Lakes, 35°53′52.10″ N 14°23′50.89″ E 25.VII.2021, with *Lasius lasioides,* leg. T. Cassar.
*Pselaphochernes lacertosus* (L. Koch, 1873)	Malta: 1 tritonymph, Valletta, 35°53′42.57″ N 14°30′38.54″ E, 9.V.2020, with *Tetramorium caespitum,* leg. T. Cassar; 1♀, Rabat, Chadwick Lakes, 35°53′52.10″ N 14°23′50.89″ E 25.VII.2021, with *Lasius lasioides,* leg. T. Cassar.
Arachnida: Trombidiformes	
*Microcaeculus* sp.	Malta: Mellieћa, Rdum Tal-Madonna, 35°59′20.54″ N 14°22′28.41″ E, 30.V.2021, with *Camponotus barbaricus,* leg. T. Cassar.
Malacostraca: Isopoda	
*Platyarthrus lerinensis* Vandel, 1957	None.
*Leptotrichus naupliensis* (Verhoeff, 1901)	None.
*Leptotrichus panzeri* (Audouin, 1826)	Malta: 6 imm. & 2♀♀, Marsa, 35°52′36.33″ N 14°29′23.72″ E, 28.VII.2021, with *Tapinoma nigerrimum,* leg. T. Cassar; Gozo: 1♂, Rabat, 36°2′25.34″ N 14°14′39.48″ E, 28.II.2020, with *Messor capitatus,* leg. T. Cassar.
Insecta: Coleoptera	
*Kissister minimus* (Laporte, 1840)	Malta: 6 exs., Mellieћa, Rdum Tal-Madonna, 35°59′20.54″ N 14°22′28.41″ E, 17.IV.2020, with *Tapinoma simrothi,* leg. T. Cassar.
Scydmaeninae gen. sp.	Malta: 1 ex., Wardija Hilltop, 35°56′13.923″ N 14°23′10.659″ E, 4.VI.2019, with *Camponotus barbaricus*, leg. A. Lapeva-Gjonova; 5 exs., Imselliet Valley, l/o Żebbiegћ and Bidnija, 35°55′4.84″ N 14°24′24.41″ E, 22.XI.2021, with *Camponotus,* leg. T. Cassar.
*Micranops* sp./*Scopaeus* sp.	Malta: 1 ex., Imselliet Valley, l/o Żebbiegћ and Bidnija, 35°55′4.84″ N 14°24′24.41″ E, 22.VII.2021, with *Linepithema humile,* leg. T. Cassar.
*Stenosis melitana* Reitter, 1894	Malta: 1♂ & 1♀, Mellieћa, Rdum Tal-Madonna, 35°59′20.54″ N 14°22′28.41″ E, 17.IV.2020, with *Tapinoma simrothi,* leg. T. Cassar.
*Symbiotes* sp.	Malta: 43 exs., Ta’ Qali, 13.II.2000, under bark of dead *Acacia* tree, leg. D. Mifsud; 1 ex., Ħal Farruġ (l/o Qormi), 26.II.2003, under bark of *Ceratonia siliqua*, leg. D. Mifsud, 1 ex., Mdina, 17.II.2000, under pigeon dung, leg. D. Mifsud.
Insecta: Diptera	
*Chrysotoxum intermedium* Meigen, 1822	None.
Insecta: Hemiptera	
*Reptalus* cf. *panzeri* (Löw, 1883)	None.

^1^ Refer to discussion Section 4.3.
